# Relationship between fibroblast growth factor in plasma and carotid plaque neovascularization: a pilot study

**DOI:** 10.3389/fimmu.2024.1385377

**Published:** 2024-04-22

**Authors:** Mahtab Zamani, Karolina Skagen, Beate Lindberg, Vigdis Bjerkeli, Pål Aukrust, Bente Halvorsen, Mona Skjelland

**Affiliations:** ^1^ Department of Neurology, Oslo University Hospital, Oslo, Norway; ^2^ Institute of Clinical Medicine, University of Oslo, Oslo, Norway; ^3^ Department of Cardiothoracic Surgery, Oslo University Hospital, Oslo, Norway; ^4^ Research Institute of Internal Medicine (RIIM), Oslo University Hospital, Oslo, Norway; ^5^ Section of Clinical Immunology and Infectious Diseases, Oslo University Hospital, Oslo, Norway

**Keywords:** atherosclerosis, unstable carotid artery plaque, intraplaque neovascularization (IPN), superb microvascular imaging (SMI), fibroblast growth factor-23, ischemic stroke, intraplaque hemmorhage

## Abstract

**Background:**

Unstable atherosclerotic carotid plaques with intraplaque neovascularization (IPN) carry a substantial risk for ischemic stroke. Conventional ultrasound methods fall short in detecting IPN, where superb microvascular imaging (SMI) has emerged as a promising tool for both visualizing and quantification. High levels of fibroblast growth factor 23 (FGF-23) have, in observational studies, been suggested as related to cardiovascular morbidity and mortality. The association of FGF-23 to atherosclerotic carotid plaque instability remains relatively unexplored.

**Methods:**

A cohort of twenty-nine patients with ≥50% atherosclerotic carotid stenosis underwent conventional carotid ultrasound, SMI, and blood tests, including measurement of FGF-23 in plasma. Nineteen patients were characterized as symptomatic and ten as asymptomatic.

**Results:**

Our major findings were: i) Higher FGF-23 levels were strongly correlated with increased SMI-assessed IPN. ii) Neo-vessel count recorded by quantitative SMI was positively correlated to increased FGF-23 levels, but not with basic FGF levels. (iii) In contrast, traditional risk factors for plaque instability exhibited no noteworthy associations with SMI-assessed IPN or with FGF-23 levels.

**Conclusion:**

This pilot study suggest the potential of FGF-23 as a valuable marker for neovascularization and atherosclerotic carotid plaque instability as a risk factor for ischemic stroke. Further research involving larger cohorts and prospective data is necessary to understand FGF-23’s role in this context comprehensively.

## Introduction

Ischemic stroke due to thromboembolism from an unstable atherosclerotic carotid plaque accounts for 15-25% of all ischemic strokes (1). Intraplaque neovascularization (IPN) is a feature of plaque vulnerability associated with an increased risk of lesion rupture and subsequent ischemic stroke (2). Therefore, identifying carotid plaques with IPN is crucial in treatment alternatives targeting stroke prevention. Pathological IPN is the sprouting of newly formed immature and leaky blood vessels from pre-existing vasa vasorum vasculature, extending throughout the entire arterial wall and towards the plaque core (3). This neovascularization is thought to occur in response to increased oxygen and nutrition demands due to inflammation and increased metabolic activity in a chronic atherosclerotic lesion (4). Still, the mechanisms leading to IPN are not fully understood, and detecting of these micro-vessels with small blood flow signals using standard Doppler ultrasound methods is challenging.

In our recent study, we introduced a novel ultrasound method, Superb Microvascular Imaging (SMI), which utilizes an algorithm that effectively overcomes the challenges faced by standard ultrasound in the visualization and quantification of IPN. We demonstrated SMI to be comparable to contrast-enhanced ultrasound for the assessment of IPN (5).

Fibroblast growth factor (FGF)-23 is a bone-secreted hormone, involved in phosphate homeostasis in the kidney and vitamin D metabolism (6). FGF-23 regulates the expression of its co-receptor, Klotho; together as a collective unit, they assemble into a trimeric signaling complex alongside FGF-receptors (FGFRs) within target tissues, facilitating the execution of FGF-23’s physiological as well pathophysiological functions. Klotho is highly expressed in renal tubules, where it downregulates sodium-phosphate cotransporters (7). Elevated FGF-23 is an independent risk factor for end-stage renal disease in patients with relatively preserved kidney function and for mortality across the spectrum of chronic kidney disease (CKD) (8). However, serum levels of FGF-23 have also been associated with a higher risk of cardiovascular disease (CVD), such as myocardial infarction, ischemic stroke, and heart failure, and these associations were not restricted to patients with impaired kidney function (9, 10). Indeed, in a population-based study, individuals with increased FGF-23 levels had a more significant burden of carotid atherosclerosis independent of CKD (11).

While increased plasma levels of FGF-23 have previously been associated with increased intima-media thickness in the common carotid artery, data on FGF-23 concerning carotid plaque instability are scarce or lacking (7). Based on its role in atherosclerosis, we hypothesized that plasma levels of FGF-23 are associated with the presence of IPN and plaque instability, as measured by SMI assessments. In this pilot study we tested this hypothesis in 29 patients with carotid atherosclerosis previously included in our SMI study cohort who had available plasma for growth factor analysis.

## Materials and methods

### Study population

We included 29 consecutive patients attending the Department of Neurology, Oslo University Hospital. Sixteen patients with available plasma from our SMI cohort study up to January 2019 were included and 13 new patients were included from January to April 2019. All patients had carotid artery plaque with stenosis ≥ 50% and underwent conventional Doppler ultrasound and SMI ultrasound of the carotid arteries before carotid endarterectomy (CEA) or at a routine outpatient control for the asymptomatic patients. Nineteen included patients were symptomatic, i.e., the patients had undergone ipsilateral cerebral ischemia (minor ischemic strokes, transitory ischemic attacks, or amaurosis fugax) within 30 days prior to study inclusion. Exclusion criteria were ongoing infection, cancer, or autoimmune disease.

The study protocol conforms to the ethical guidelines of the 1975 Declaration of Helsinki. The study was approved by the Norwegian Regional Committee for Medical and Health Research Ethics (ID REC 2014/1468), and written informed consent was obtained from all patients before study inclusion.

### Ultrasonographic investigation

Ultrasonography Imaging was performed with a Canon ultrasound system Aplio 500 (Canon Medical Systems, Otawara, Japan) using a 7.5 MHz linear probe on both carotid arteries for standard Doppler ultrasound and SMI ultrasound. Common carotid artery, carotid bifurcation, and internal carotid arteries were examined in longitudinal and transverse planes in standard ultrasound. The degree of carotid artery stenosis was determined based on peak-systolic and end-diastolic velocities according to consensus criteria of the Society of Radiologists in ultrasound (12). Plaque echogenicity was classified visually in high-resolution B-mode gray-scale pictures according to the modified version of the classification proposed by Gray-Weale classification as follows: (1) uniformly or predominantly hypoechoic (hypoechoic or hypoechoic with small hyperechoic regions), (2) uniformly or predominantly hyperechoic (hyperechoic or hyperechoic with small hypoechoic regions) (13).

### Superb microvascular imaging

Following the standard ultrasound examination, the monochrome-SMI mode was turned on, twin-view display of the plaque in B-mode and monochrome-SMI side by side was enabled. Details on optimized SMI settings and configurations for the assessment of IPN is previously published (5). Plaques were first observed in the transverse plane and then in the longitudinal plane for 2 minutes, and the video images were stored in the ultrasound scanner’s hard drive. Static enhancements were excluded, and moving enhancements were classified as intraplaque microvascular flow (IMVF). IMVF signals were first categorized into three groups on a visual scale as follows: No IPN (no IMVF within the plaque or IMVF confined to the adjacent adventitia), moderate IPN (moving IMVF confined to the adventitial side or moving IMVF at the plaque shoulder), and extensive IPN (IMVF moving to the plaque core or expanded IMVF throughout plaque). Second, a visual assessment of IMVF signals involved tallying the number of neovessels observed within a 2-minute SMI video clip, providing a quantitative evaluation of IPN when assessed using SMI (5).

### Biochemical investigation

Blood samples were analyzed by routine methods at the Department of Clinical Biochemistry, Oslo University Hospital. They included Low-Density Lipoprotein (LDL) cholesterol, High-Density Lipoprotein (HDL) cholesterol, Triglycerides (TG), Total cholesterol, fasting glucose, glycosylated Hemoglobin (HbA1c), estimated Glomerular filtration rate (eGFR), high-sensitivity C-reactive protein (CRP), Leukocyte and platelets count, sedimentation rate (ESR) and electrolytes. In addition, EDTA plasma was collected and kept on melting ice for 30 minutes until centrifuged at 2000*g* for 20 minutes to obtain platelet-poor plasma. Plasma was stored at -80°C until FGF-23, basic (b)-FGF, Interleukin-6 (IL-6) and Tumor Necrosis Factor alpha (TNF-α) were analyzed by U-Plex Metabolic Group 1 (human) assay (Meso Scale Diagnostics, Rockville, MD, USA).

### Statistical methods

SPSS for Windows statistical software (version 28.0) was used for data analyses. Categorical variables were compared with Kruskal Wallis and *post hoc* pairwise comparison tests were used to perform the statistical analysis. Coefficients of correlation were calculated by the Spearman ρ correlation for scale variables. The factors found significant were included in the linear multiple regression model. All statistical results were considered significant when *p*<0.05.

## Results

Twenty-nine patients participated in this study, 18 [70 ± 6 years] men and 11 women [77 ± 6 years] (**Table 1**). Plaque characteristics assessed by conventional ultrasound and advanced ultrasound assessment of IPN are presented in **Table 2**. Eleven (38%) uniformly or predominantly hypoechoic and 18 (62%) uniformly or predominantly hyperechoic plaques were examined with B-mode ultrasound. SMI revealed IMVF signal representing IPN in 23 of the 29 plaques assessed visually and quantitatively. Of the 23 patients exhibiting IPN on SMI, nine patients (31%) exhibited moderate IPN, and 14 patients (48%) displayed extensive IPN on the visual scale.

**Table 1:** Baseline variables of the study population.

	Patients N=29
Age, (years)^a^	72.5 (6.8)
Male sex	18 (62.1)
Current or former smoke	13 (44.8)
Hypertension	18 (62.1)
Diabetes mellitus	4 (13.1)
Dyslipidemia	13 (44.8)
Cerebrovascular symptoms	19 (65.5)
Chronic kidney disease	3 (10.3)
Coronary artery disease	11 (37.9)
Aspirin treatment	21 (72.4)
Statin treatment	25 (86.2)
CRP, (mg/L)^a^	5.3 (8.6)
Total cholesterol, (mM/l)^a^	5.6 (9.1)
LDL cholesterol, (mM/l)^a^	2.3 (1.3)
HDL cholesterol, (mM/l)^a^	2.6 (6.8)
Triglycerides, (mM/l)^a^	2.7 (7.3)
LDL/HDL ratio	1.8 (1.1)
Creatinin^a^	25 (86.2)
eGFR ml/min/1.73 m2	73.6 (16.4)
HbA1c, (%)^a^	6.2 (1.5)

Data are given as number (percentages) or ^a^ mean (SD). HDL, high-density lipoprotein; LDL, low-density lipoprotein; eGFR, estimated Glomerular filtration rate.

**Table 2:** Plaque characteristics using standard Doppler ultrasound, IPN assessment using SMI (N=29).

**Degree of stenosis**	50-69% >70%	6 (20.7) 23 (79.3)
**Plaque echogenicity**	Predominantly hypoechoic Predominantly hyperechoic	11 (37.9) 18 (62.1)
**SMI-count, quantitative** ^a^		4.5 (4.4)
**IMVF signal on SMI, visual semiquantitative scale**	No IPN Moderate IPN Extensive IPN	6 (20.7) 9 (31) 14 (48.3)

Data are given as number (percentages) or ^a^ mean (SD). IMVF, Intraplaque microvascular flow. IPN, Intraplaque neovascularization.

### Correlation between plasma level FGF-23 and IPN

**Figure 1:**
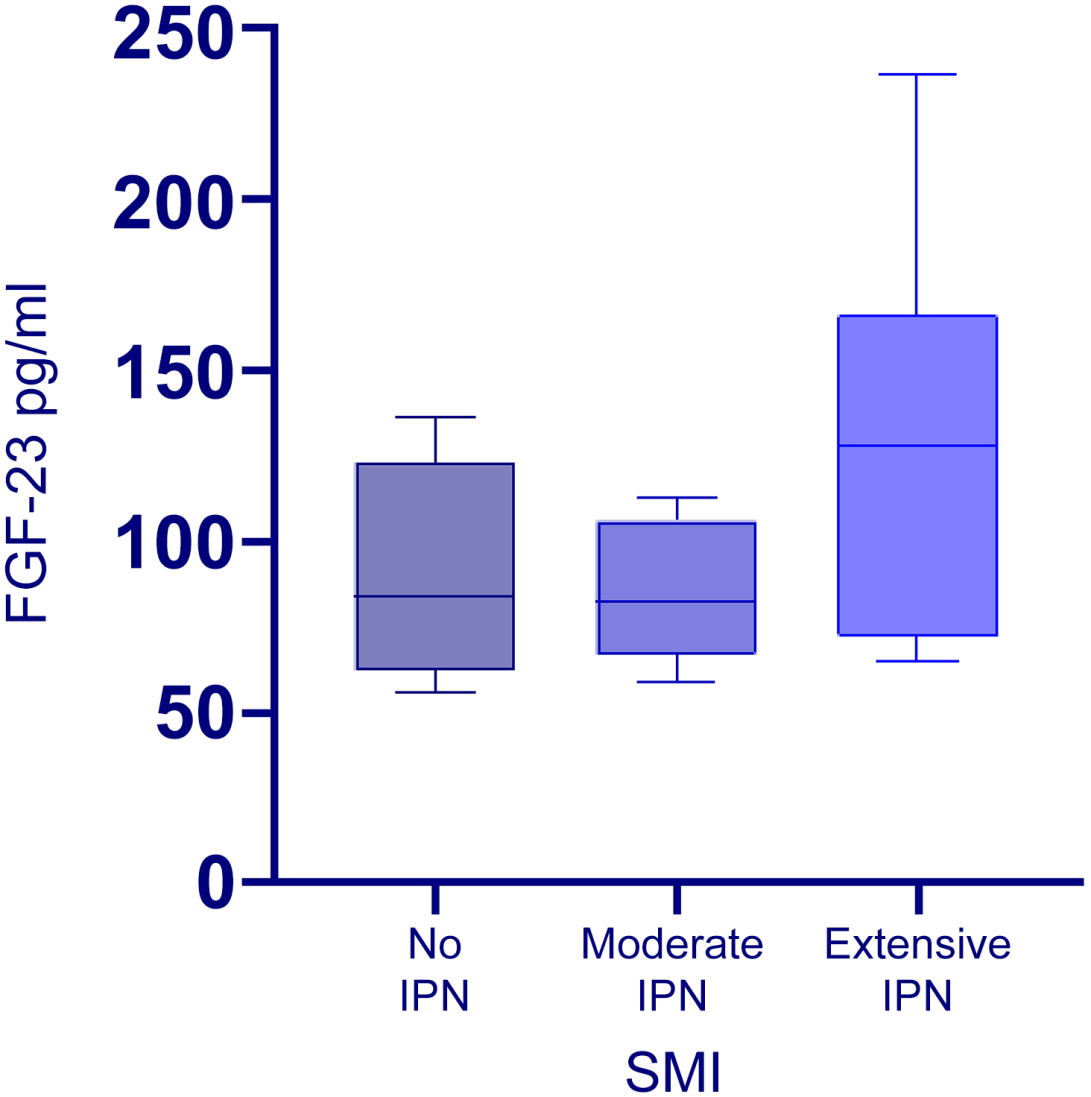
Box plot showing median serum level FGF-23 distribution in patients with no IPN, moderate IPN, and extensive IPN on SMI. Horizontal lines in boxes represent the median levels (second quartile), and the whiskers are the range limits. The median plasma level FGF-23 was respectively 84.2 (range 55.8-136.2), 78.1 (range 58.5-112.7) and 132.8 (range 64.9-236.5).

**Figure 2:**
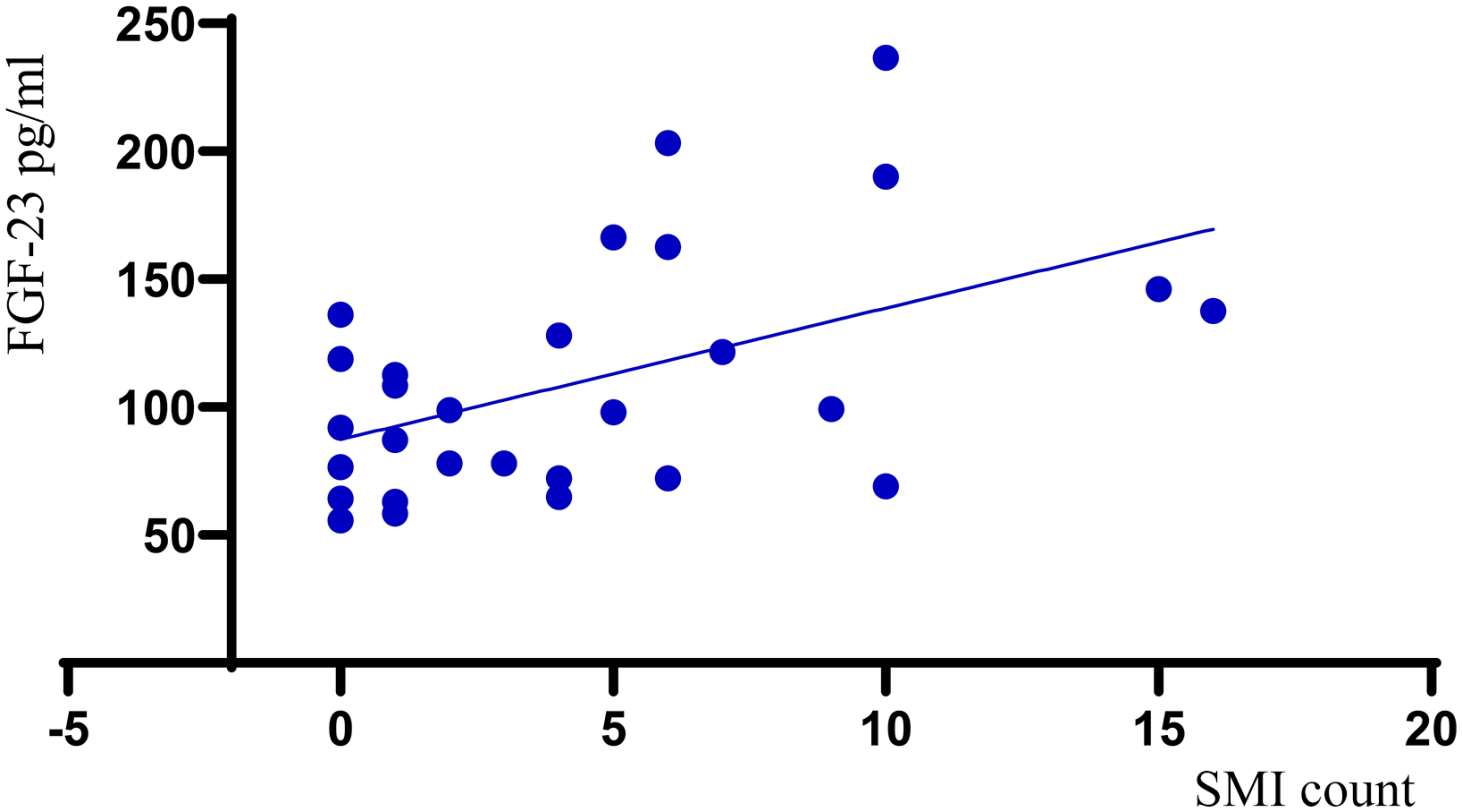
Scatter plot demonstrating the relationship between FGF-23 values and quantitative SMI (SMI count).

Interestingly, plaques with higher grades of IMVF signal on the semi-quantitative-SMI visual scale had significantly higher values of circulating FGF-23 levels (*p*=0.011, r=0.466) (**Figure 1**). Neo-vessel count recorded by quantitative SMI was also positively correlated to higher circulatory FGF-23 levels (*p*=0.007, r= 0.491) (**Figure 2**). Patients with lower eGFR had statistically higher plasma level FGF-23 (*p*=0.030, r= -0.347) and higher neo-vessel counts on SMI (*p*= 0.030, r=-0.403). Including FGF-23 and eGFR, regression analyses indicated that the model explained 30% of the variance and was a significant predictor of intraplaque neo-vessel count assessed by SMI, F (2.26) = 5.5, *p*< 0.001. While plasma level of FGF-23 alone significantly predicted SMI-assessed intraplaque neo-vessel count (β= 0.389, *p*= 0.04), eGFR did not (β= -0.256, *p*=0.163).

We next employed the Kruskal-Wallis test to assess whether there are significant differences in FGF-23 values across the three visual SMI classification groups. Whereas the Kruskal-Wallis test yielded a significant result (H=7.23, df = 2, *p*= 0.027), the *post hoc* pairwise comparison revealed significant differences in FGF-23 values between patients with moderate IPN and those with extensive IPN (*p*= 0.016) and trend for significant differences between no IPN and extensive IPN groups (*p*=0.052). These results further support higher values of FGF-23 among patients with extensive IPN assessed with SMI compared to the other groups. There was no differences in baseline variables in the three SMI IPN groups (**Supplementary Table S1**).

In contrast to FGF-23, there was no significant relation between b-FGF and quantitative and semi-quantitative SMI-assessed IPN (*p*=0.598, r=0.106 and *p*=0.577, r=-0.084, respectively).

To further analyze if the associations of FGF-23 and IPN merely reflect activation of inflammatory pathway, we analyzed the levels of IL-6 and TNF-α, two prototypical inflammatory cytokines. However, in contrast to FGF-23, plasma levels of these cytokines showed no correlation with quantitative and semi-quantitative SMI-assessed IPN (*p*=0,482, r=-0.136, *p*=0.370, r=-0.173 and *p*=0,723, r=0.069, *p*= 0.765, r=0.058, respectively).

### No correlation between plasma FGF-23 and traditional cardiovascular risk factors

No statistically significant relationship was found between plasma level FGF-23 and traditional risk factors for CVD (hypertension, hypercholesterolemia, diabetes mellitus, and smoking) or plasma levels of total cholesterol, HDL/LDL ratio, Triglycerides, CRP. (**Supplementary Table S2**). Moreover, in contrast to FGF-23 these risk factors were not associated with quantitative and semi-quantitative SMI-assessed IPN (**Supplementary Table S2**).

## Discussion

In this pilot study we investigated the association between plasma level FGF-23 and atherosclerotic carotid plaque IPN assessed by SMI both semi-quantitatively and quantitatively. Our main and novel findings were: i) plaques with higher grades of IMVF signal on semi-quantitative SMI visual scale assessment of IPN, had significantly increased levels of circulating FGF-23 levels, ii) patients in Extensive-IPN group assessed with semi-quantitative SMI had increased plasma levels of FGF-23 compared to the other patient groups (No-IPN group and Moderate-IPN group) iii) Neo-vessel count recorded by quantitative SMI was positively correlated to increased plasma level FGF-23 levels and this association remained significant after adjusting for eGFR iv) FGF-23 did not correlate with traditional risk factors for CVD, and in contrast to FGF-23, these risk factors did not correlate with SMI-assessed IPN.

An association between circulatory FGF-23 and unstable carotid plaque has previously been reported by Biscetti et al. in patients with type two diabetes mellitus, using histological assessment of plaque after endarterectomy (14). To the best of our knowledge, the current report is the first to demonstrate increased plasma level FGF-23 to be independently associated with increased SMI-assessed IPN, an essential sign of plaque instability in a population with carotid atherosclerosis. Importantly, in the current cohort, only 13.8% had diabetes, and 10.3% had CKD. The association of FGF-23 with SMI-assessed IPN was also seen after adjusting for eGFR. In contrast, other risk markers for plaque instability, such as an unfavorable risk profile and systemic inflammation—as assessed by CRP and prototypical cytokines TNF-α and IL-6—were not associated with SMI-assessed IPN.

Previous studies have shown that FGF-23 is an independent predictor of cardiovascular events in the community (11, 15, 16), and even in a population at high risk of CVD with intact phosphate-FGF-23-calcitriol system (10) raising the question of whether circulating FGF-23 may reflect novel and important aspects of cardiovascular risk yet to be unraveled and may possibly be involved in vascular pathology. Nirav et al. showed a clear relationship between FGF-23 and the presence of atherosclerotic plaque and size, studying 1512 stroke-free participants with FGF-23 serum levels and 2D carotid ultrasound data available (11). There were, however, no records of the degree of stenosis or hemodynamic effect of plaques nor any assessment of plaque morphology. In another study, Rodríguez-Ortiz et al. showed that increased vessel wall intima-media thickness (IMT) was significantly associated with higher median serum level FGF-23 (10); however, IMT measurements were done at common carotid arteries. It is well known that the most prevalent site for pre-cranial carotid artery atherosclerosis is at the carotid artery bifurcation alongside the proximal internal carotid artery (17). To our knowledge, the present study is the first to show higher levels of plasma FGF-23 in patients with higher SMI neo-vessel count and IPN visual grades as a sign of plaque instability, more clearly linking FGF-23 levels to plaque destabilization of carotid atherosclerosis.

Pathophysiology of atherosclerosis is a complex interaction of cellular elements (e.g., platelets, monocytes/macrophages, lymphocytes, endothelial cells, and smooth muscle cells) acting locally and systemically involving a bidirectional interaction between lipids and inflammation. Whereas most previous studies on FGF-23 are performed on subclinical carotid plaques or without complete ultrasonographic examination, the present study suggests that FGF-23 could be one of the contributing factors to plaque progression and induction of plaque instability. Notably, we found no association between basic FGF (b-FGF) and SMI-assessed IPN, suggesting that this property is more specific for FGF-23.

The mechanisms by which FGF-23 may induce plaque instability are not clear. However, Klotho, the FGF-23 obligate co-receptor, is expressed in human vascular tissue (18), and both Klotho and FGF-23 regulate vascular tone (19). Moreover, although FGF-23 displays the highest expression in bone, it can also be detected in other organs, including the brain, heart, intestine, and skeletal muscle (20), and notably, the FGF receptors, FGFR1 and FGFR4 are expressed in tunica media of several arteries and veins including heart microvessels (21). Moreover, Del Porto et al. detected FGF-23 inside the atherosclerotic plaque after endarterectomy and plaque immunohistochemistry (22).

The main limitation of this study is the sample size, which is too small to make any firm conclusion. Moreover, although the association of FGF-23 and IPN was not seen for CRP a reliable marker of general inflammation and prototypical cytokines IL-6 and TNF-α, we cannot exclude that other molecules related to inflammation and fibrogenesis could be of more importance than FGF-23. Forthcoming studies should therefor include the analyses of other potential markers that could be related to IPN as well as analyses within the plaques. The strengths are the well-clinically defined group, including the advanced and accurate carotid ultrasound examinations.

## Conclusion

This pilot study adds knowledge on the association between plasma FGF-23 levels and atherosclerotic carotid plaque neovascularization, underscoring the potential of FGF-23 as a valuable marker for neovascularization and atherosclerotic carotid plaque instability and a risk factor for ischemic stroke. Further research, including larger validation cohorts and prospective data about clinical outcomes, is needed to clarify the role of FGF-23 as a marker and potentially also a mediator and novel therapeutic target in carotid atherosclerosis.

## Data availability statement

The raw data supporting the conclusions of this article will be made available by the authors, without undue reservation.

## Ethics statement

The studies involving humans were approved by Norwegian Regional Committee for Medical and Health Research Ethics (ID REC 2014/1468). The studies were conducted in accordance with the local legislation and institutional requirements. The participants provided their written informed consent to participate in this study.

## Author contributions

MZ: Conceptualization, Data curation, Formal analysis, Investigation, Methodology, Visualization, Writing – original draft, Writing – review & editing. KS: Conceptualization, Formal analysis, Methodology, Supervision, Validation, Writing – review & editing. BL: Investigation, Writing – review & editing. VB: Formal analysis, Resources, Writing – review & editing. PA: Conceptualization, Methodology, Supervision, Validation, Visualization, Writing – review & editing. BH: Conceptualization, Formal analysis, Resources, Visualization, Writing – review & editing. MS: Conceptualization, Data curation, Formal analysis, Funding acquisition, Methodology, Project administration, Resources, Supervision, Validation, Writing – review & editing.
