# Supplementary material for: Relationship between fibroblast growth factor in plasma and carotid plaque neovascularization: a pilot study
Source: Front Immunol. 2024 Apr 22;15:1385377. doi: 10.3389/fimmu.2024.1385377 (PMC11070475; doi:10.3389/fimmu.2024.1385377)
Supplement: Supplementary file 1 [file Table_1.docx]

**Supplemental Table 1.** Baseline variables in the three semi-quantitative SMI IPN groups.

|  | **No IPN**  **(N=6)** | **Moderate IPN**  **(N=9)** | **Extensive IPN**  **(N=14)** | **p** |
| --- | --- | --- | --- | --- |
| Male gender | 4 (66.7) | 3 (33.3) | 8 (57.1) | 0.64 |
| Age, years * | 72 (8.4) | 70 (5.7) | 74 (6.6) | 0.33 |
| Cholesterol mmol/l * | 4 (0.69) | 3.7 (1) | 7.6 (13.1) | 0.47 |
| HDL/LDL ratio * | 1.9 (1.1) | 1.6 (1) | 1.8 (1.2) | 0.77 |
| Triglycerides mmol/l * | 1.2 (0.5) | 1.3 (0.6) | 4.3 (10.5) | 0.62 |
| CRP mg/L* | 9.5 (12.3) | 2.6 (2.2) | 5.3 (9.2) | 0.22 |
| eGFR ml/min/1,73m2 * | 78 (8.7) | 80.2 (15.3) | 67.4 (18.1) | 0.10 |
| Hypertension | 5 (83.3) | 7 (77.8) | 12 (85.7) | 0.64 |
| Statin Treatment | 6 (100) | 6 (66.7) | 13 (92.9) | 0.81 |
| Diabetes mellitus | 0 (0) | 1 (11.1) | 3 (21.4) | 0.46 |
| Current or former smoker | 4 (66.7) | 7 (77.8) | 6 (42.9) | 0.46 |

Data is given as numbers (percentages) or mean (SD). HDL, high-density lipoprotein; LDL, low-density lipoprotein; CRP, C-reactive protein; eGFR, estimated Glomerular filtration rate.

**Supplemental Table 2.** Correlation between FGF-23 and traditional cardiovascular risk factors and between SMI assessed IPN and cardiovascular risk factors.

|  | **FGF-23** | **Quantitative-SMI** | **Semi-quantitative-SMI** |
| --- | --- | --- | --- |
| Hypertension | r= -0.251, *p*= 0.189 | r= 0.066, *p*= 0.734 | r= -0.053, p=0.784 |
| Statin Treatment | r= 0.036, *p*= 0.857 | r= -0.145, *p*=0.454 | r= 0.050, p= 0.805 |
| Diabetes mellitus | r= 0.137, *p*= 0.477 | r= -0.145, *p*=0.454 | r= -0.240, p= 0.210 |
| Smoking | r= -0.197, *p*= 0.307 | r= -0.253, *p*= 0.185 | r= -0.263, p= 0.168 |
| Cholesterol mmol/l | r= -0.264, *p*= 0.167 | r= 0.115, p=0.553 | r= 0.074, p= 0.704 |
| HDL/LDL ratio | r= 0.091, *p*= 0.635 | r= -0.185, p=0.336 | r= -0.080, p= 0.681 |
| Triglycerides mmol/l | r= -0.017, *p*= 0.929 | r= 0.190, p=0.323 | r= 0.182, *p*= 0.345 |
| CRP mg/L | r= 0, *p*= 0.999 | r= -0.340, *p*= 0.071 | r= -0.224, *p*= 0.242 |

Data are given as Spearman rho (r) and *p*
